# Supplementary material for: Change in the Results of Motor Coordination and Handgrip Strength Depending on Age and Body Position—An Observational Study of Stroke Patients and Healthy Volunteers
Source: Int J Environ Res Public Health. 2022 Apr 13;19(8):4703. doi: 10.3390/ijerph19084703 (PMC9026978; doi:10.3390/ijerph19084703)
Supplement: Supplementary file 1 [file ijerph-19-04703-s001.zip › ijerph-1634845-supplementary.pdf]

Table S1. Assessment of differences in biometric data in the studied groups.

Age		Stroke	Control	Z	*p*	Effect Size
18–45	Height	175.20 ± 9.88	173.80 ± 8.14	−0.32	0.841	0.10
	Body mass	71.20 ± 9.88	68.80 ± 11.08	−0.41	0.690	0.13
	BMI	23.10 ± 1.38	22.63 ± 1.69	−0.73	0.548	0.23
46–60	Height	170.42 ± 5.68	167.17 ± 7.03	−1.24	0.219	0.25
	Body mass	75.50 ± 7.30	71.17 ± 11.34	−1.13	0.266	0.23
	BMI	25.97 ± 1.82	25.56 ± 4.56	−1.24	0.219	0.25
>61	Height	170.67 ± 9.00	166.63 ± 7.80	−1.19	0.234	0.13
	Body mass	77.07 ± 10.16	73.75 ± 10.92	−1.10	0.273	0.12
	BMI	26.40 ± 2.37	26.53 ± 3.25	−0.30	0.767	0.03

U Mann-Whitney test

Table S2. Assessment of differences in biometric data in the studied age ranges.

Age		Stroke	Control	*p* [a]	φ
18–45	Gender				
	Female	1	2	1.000	0.22
	Male	4	3		
46–60	Gender				
	Female	3	5	0.667	0.18
	Male	9	7		
>61	Gender				
	Female	26	25	1.000	0.02
	Male	17	15		

[a]—*p*-value of Fisher exact test

Table S3. Assessment of differences in TCT and FMA UE results in the studied age ranges of patients after stroke.

Age		Stroke	Control	Z	*p*	Effect Size
18–45	TCT	89.80 ± 13.97	100.00 ± 0.00	−1.49	0.310	0.47
	FMA-UE	46.20 ± 2.59	66.00 ± 0.00	−2.78	**0.008**	0.88
46–60	TCT	80.67 ± 11.66	100.00 ± 0.00	−3.64	**0.001**	0.74
	FMA-UE	46.17 ± 2.08	66.00 ± 0.00	−4.45	**<0.001**	0.91
>61	TCT	82.74 ± 12.14	100.00 ± 0.00	−6.26	**<0.001**	0.67
	FMA-UE	45.67 ± 2.05	66.00 ± 0.00	−8.33	**<0.001**	0.91

U Mann-Whitney test

Table S4. Assessment of differences in MAS results in the studied age ranges of patients after stroke.

Age	MAS	Stroke	Control	p [a]	φ
18–45	0	0	5		
	1	3	0	**0.008**	1.00
	+1	2	0		
46–60	0	0	12		
	1	8	0	**<0.001**	1.00
	+1	4	0		
>61	0	0	40		
	1	27	0	**<0.001**	1.00
	+1	16	0		

[a]—p-value of Fisher exact test

Table S5. There are no differences between age groups among patients after stroke in terms of the following parameters: TCT, FMA UE, MAS.

	H	p	η2	V
TCT	2.56	0.278	0.01	N/A
FMA-UE	0.7	0.704	< 0.01	N/A
MAS	N/A	1.000	N/A	0.04
